# Adjuvant Chemotherapy and Tumor Sidedness in Stage II Colon Cancer: Analysis of the National Cancer Data Base

**DOI:** 10.3389/fonc.2020.568417

**Published:** 2020-09-15

**Authors:** Shiva Kumar R. Mukkamalla, Donny V. Huynh, Ponnandai S. Somasundar, Ritesh Rathore

**Affiliations:** ^1^Division of Hematology/Oncology, Presbyterian Medical Group, Rio Rancho, NM, United States; ^2^McLeod Oncology and Hematology Associates at Seacoast, Little River, SC, United States; ^3^Division of Surgical Oncology, Roger Williams Medical Center/Boston University School of Medicine, Providence, RI, United States; ^4^Division of Hematology/Oncology, Roger Williams Medical Center/Boston University School of Medicine, Providence, RI, United States

**Keywords:** colorectal cancer, stage 2, adjuvant chemotherapy, tumor sidedness, national cancer data base

## Abstract

**Background:** Current guidelines recommend discussion of adjuvant chemotherapy (AC) for stage II colon cancer (CC) with high-risk features despite lacking conclusive randomized trial data. We examined AC administration in this population and its effect on overall survival (OS) for available patient, tumor, and treatment characteristics

**Methods:** Using National Cancer Data Base, a cohort of 42,971 stage II CC patients diagnosed from 2004 to 2009, who underwent surgery with curative intent, was identified. Chi-square test and multivariate logistic regression were used to analyze baseline characteristics and to calculate odds of chemotherapy administration, respectively. Survival analysis was conducted using Kaplan Meier survival analysis with log-rank test and Cox proportional hazards regression modeling.

**Results:** AC was administered to 26% patients. The use decreased with advancing age and elderly patients received more single-agent than multi-agent chemotherapy (3 vs. 2.4%, *p* < 0.0001). Major predictors of AC use included pT4 status, evaluation of <12 lymph nodes, high grade tumors, positive resection margins, age <65 years, left sided tumors, and low comorbidity score. AC was associated with improved OS regardless of high-risk features (pT4, undifferentiated histology, <12 lymph node evaluation, or positive resection margins), tumor location, age, gender, comorbidity index, chemotherapy regimen or type of colectomy (adjusted HR: single-agent 0.55, multi-agent 0.6; *p* < 0.0001). In subgroup analysis, AC use compensated for the survival differences otherwise seen between left and right sided tumors in the non-chemotherapy population.

**Conclusion:** AC in stage II CC was associated with improved OS regardless of age, chemotherapy type or high-risk features. It improved 5-years OS irrespective of tumor location and seemed to compensate for the survival difference seen between right and left sided tumors noted in the non-chemotherapy group.

## Introduction

Colorectal cancer is the third leading cause of cancer diagnosis in the United States (U.S.), both among men and women (1, 2). It is also the second most common cause of cancer death when men and women are combined. As of January 2019, it was estimated that there were in excess of 1.5 million patients in the U.S. with a diagnosis of colorectal cancer (3). In 2020, it is estimated that an additional 147,950 new cases and 53,200 deaths will occur in the U.S (2). Surgical resection remains the mainstay of treatment for non-metastatic colon cancer, with adjuvant chemotherapy having demonstrated improved overall survival (OS) for stage III colon cancer patients (4–7). In patients with stage II colon cancer, the role of adjuvant chemotherapy remains a point of debate (8–16). Since there are few definitive prospective clinical trials which have evaluated the use of adjuvant chemotherapy in stage II colon cancer, current clinical practice guidelines by the American Society of Clinical Oncology (ASCO) and the National Comprehensive Cancer Network (NCCN) recommend discussing chemotherapy in patients with tumors possessing high-risk features or microsatellite stability (MSS) and all T4 tumors (17–20). Stage II microsatellite instability high (MSI-H) patients may have a good prognosis and do not benefit from 5-fluorouracil (5-FU) adjuvant chemotherapy (21). In contrast, population-based studies have failed to demonstrate substantial OS benefit with the use of adjuvant chemotherapy for all patients with poor-prognostic or high-risk features (22, 23).

Recently, a study looking at stage II colon cancer patients diagnosed from 1998 to 2006 using the National Cancer Data Base (NCDB) was able to demonstrate an OS benefit associated with adjuvant chemotherapy (24). Though the sample size in this study was large (*N* = 153,110), it included patients with other malignancies, thereby resulting in a competing mortality bias.

The primary objective of our study was to utilize data from the NCDB and assess for OS benefit of adjuvant chemotherapy in stage II colon cancer patients, with no other malignancies, diagnosed from 2004 to 2009. Since the US Food and Drug Administration (FDA) approved combination of 5-FU, leucovorin and oxaliplatin (FOLFOX) in 2004 (25), all patients receiving multi-agent chemotherapy in our study population were hypothesized to have received the FOLFOX regimen. The secondary objectives of our study were to assess the impact of tumor sidedness on OS with or without adjuvant chemotherapy, as well as to evaluate the association between adjuvant chemotherapy and the two major high-risk features of stage II colon cancer i.e., T4 tumors and inadequate lymph node evaluation (<12 lymph nodes). In addition, a multivariate analysis of determinant factors in utilization of chemotherapy from 2004 to 2009 was also performed.

## Patients and Methods

### Data Source

The NCDB is a joint quality improvement initiative of the American College of Surgeons' Commission on Cancer and the American Cancer Society. The NCDB contains 34 million patient records, and represents ~70% of all newly diagnosed cases of cancer in the United States (26, 27). Data access was approved by the NCDB after a thorough review of the study proposal. Participant User File (PUF), which included patients diagnosed with colon cancer from 2004 to 2014, was utilized to extract the study cohort.

### Study Population

**Figure 1:**
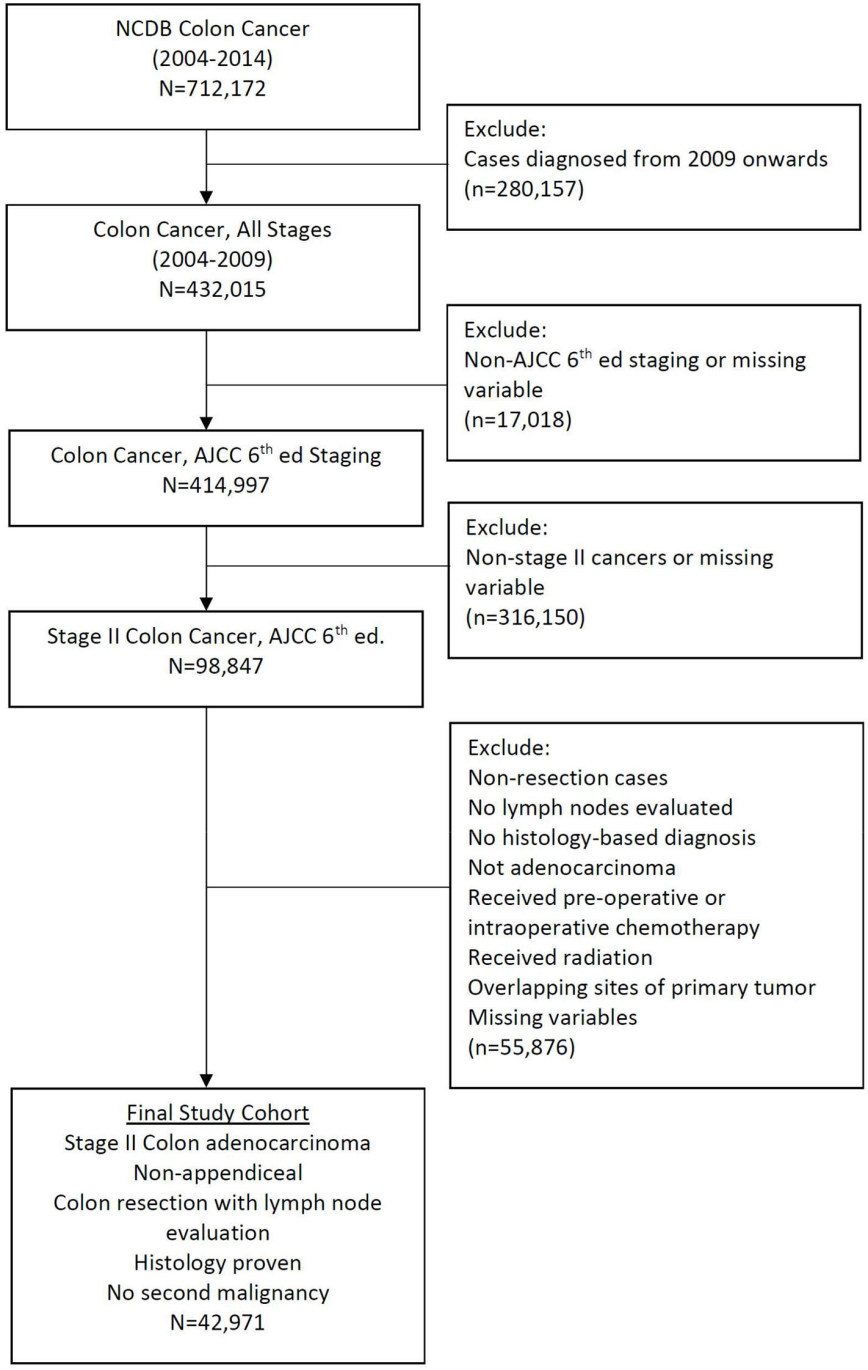
CONSORT diagram.

The American Joint Committee on Cancer (AJCC) sixth edition was used for staging purposes and site-specific information was defined according to the AJCC's Collaborative Stage Data Collection System (CCS). Using CCS we excluded patients with appendiceal adenocarcinoma along with exclusion of those who underwent surgical procedures spanning less than a partial colectomy. Only patients with a pathologically confirmed diagnosis were included for analysis. Patients lacking documentation about the variables of interest were also excluded. A final cohort of 42,971 patients diagnosed with stage II colon cancer from 2004 to 2009 was identified using an age-mandated eligibility criteria (18 years and above), along with above mentioned inclusion and exclusion criteria (Figure 1). The year 2009 was chosen as a cut-off to enable a minimum follow-up of 5 years for all patients while maintaining a uniform cancer staging system (AJCC, 6th edition).

### Measured Outcomes and Variables

The primary endpoint of this study was the 5-years OS. In patients who were alive at the last follow-up, OS was censored at 60 months. Age was analyzed as an ordinal variable after being grouped into 18–64 years, 65–74 years and above. Patients were categorized into four ethnic groups; Caucasians, African-Americans, Hispanics, and others. Patient performance status was analyzed using the Charlson/Deyo comorbidity index and the primary site of colon cancer was recoded into left, right, or transverse part of the colon. Other variables analyzed included gender, institution (academic vs. non-academic), insurance status, average neighborhood income level, year of diagnosis, geographic location of treating institution, histologic grade, involvement of margins, adequacy of lymph nodes evaluated during surgery, pathologic primary tumor characteristics (pT), type of colectomy, and adjuvant chemotherapy.

### Statistical Analysis

Descriptive analysis of patient's demographic and clinical information according to receipt of adjuvant chemotherapy was performed using the Pearson chi-square test. Multivariate logistic regression model was used to calculate the odds ratio (OR) of chemotherapy administration based on several determinant factors including age, race, baseline comorbidity index, institution, geographic location, year of diagnosis, tumor laterality, grade, adequate lymph node evaluation, pT, surgery, and margins.

Kaplan Meier survival curves and Cox proportional hazards model were utilized to perform survival analysis. Kaplan Meier survival curves were adjusted and tested with the log-rank test. A Cox proportional hazards model was constructed using age, average neighborhood income level and Charlson/Deyo comorbidity index as ordinal variables. Other variables including gender, race, institution, insurance status, year of diagnosis, location of primary tumor, histologic grade, adequacy of lymph node evaluation, pT, margins, type of surgery and adjuvant chemotherapy were analyzed as categorical variables in this model. Hazards ratios (HR) and 95% confidence intervals (CI) were generated with HR <1.0 indicating survival benefit.

The *p* < 0.05 was considered statistically significant and Statistical Analysis Software (SAS version 9.4; SAS Institute, Cary, NC) was used for all analyses.

## Results

### Patient Characteristics

We identified 42,971 patients from NCDB who were diagnosed with stage II colon cancer between 2004 and 2009. Patient and tumor characteristics are shown in Table 1, stratified according to receipt of adjuvant chemotherapy. The overall frequency of adjuvant chemotherapy administration was 26% and did not differ significantly by the academic level of treating institution, median family income level or adequacy of lymph node evaluation.

**Table 1:** Baseline patient characteristics, stratified according to receipt of adjuvant chemotherapy.

	**No chemotherapy (*n* = 33,986)**	**Chemotherapy (*n* = 8,985)**	***p***
**Age, (%)**			<0.0001
18–64 years	59	41	
65–74 years	77.1	22.9	
≥75 years	93.8	6.2	
**Gender, (%)**			<0.0001
Male	77.5	22.5	
Female	80.4	19.6	
**Race, (%)**			<0.0001
White	80	20	
Black	75.6	24.4	
Hispanic	71.9	28.1	
Others	76.6	23.4	
**Charlson/Deyo score, (%)**			<0.0001
0	76.2	23.8	
1	82.9	17.1	
2	90	10	
**Institution, (%)**			0.7356
Academic	79	21	
Non-academic	79.1	20.9	
**Location, (%)**			<0.0001
East North Central	78.6	21.4	
East South central	77.2	22.8	
Middle Atlantic	77	23	
Mountain	79.9	20.1	
New England	82.6	17.4	
Pacific	81.5	18.5	
South Atlantic	79.5	20.5	
West North Central	78.8	21.2	
West South Central	77.7	22.3	
**Insurance, (%)**			<0.0001
Insured	79.6	20.4	
Uninsured	62.4	37.6	
**Income, (%)**			0.1188
< $30,000	78.7	21.3	
$30,000–$34,999	78.3	21.7	
$35,000–$45,999	79.7	20.3	
≥$46,000	79	21	
**Year of diagnosis, (%)**			<0.0001
2004	79.5	20.5	
2005	78.9	21.1	
2006	77.6	22.4	
2007	78	22	
2008	80.1	19.9	
2009	80.6	19.4	
**Primary site, (%)**			<0.0001
Left	74.4	25.6	
Right	82.1	17.9	
Transverse	79.6	20.4	
**Grade, (%)**			<0.0001
Well-differentiated	81.1	18.9	
Moderately diff	79.7	20.3	
Poor/Undifferentiated	75.2	24.8	
**Nodes evaluated, (%)**			0.6588
Adequate (≥12)	79.1	20.9	
Inadequate (<12)	78.9	21.1	
**AJCC pT, (%)**			<0.0001
3	81.5	18.5	
4	56.5	43.5	
**Colectomy, (%)**			<0.0001
Partial	77.5	22.5	
Subtotal	80.1	19.9	
Total	75.4	24.6	
**Margins, (%)**			<0.0001
Negative	79.6	20.4	
Positive	62.8	37.2	

Very elderly patients (age ≥ 75 years) received significantly less chemotherapy as compared to the elderly (65–74 years) and young (18–64 years) patient population (6.2 vs. 22.9% vs. 41%, *p* < 0.0001). Women were less likely to receive adjuvant chemotherapy as compared to men (19.6 vs. 22.5%, *p* < 0.0001). Among Caucasians, only 20% received chemotherapy as compared to Hispanics (28.1%), African Americans (24.4%), and other ethnicities (23.4%), which was a significant difference (*p* < 0.0001). Patients with higher comorbidity index i.e., the Charlson/Deyo score, were less likely to receive adjuvant chemotherapy (10 vs. 17.1 vs. 23.8%, *p* < 0.0001). Among the nine broadly divided geographic regions in the U.S., patients in New England were least likely to receive chemotherapy (17.4%, *p* < 0.0001). Adjuvant chemotherapy administration was more common in those without insurance than with (37.6 vs. 20.4, *p* < 0.0001). Frequency of chemotherapy administration steadily increased from 2004 to 2006 (20.5–22.4%, *p* < 0.0001), but then gradually decreased to 19.4% as of 2009.

Patients with tumors located on the left side of colon more often received adjuvant chemotherapy (25.6%) compared to those with tumors of the right (17.9%) or transverse colon (20.4%), which was significantly different as depicted by *p* < 0.0001. Furthermore, patients with high-risk features including pT4 (43.5%), positive margins (37.2%), and high grade tumors (24.8%) more often received adjuvant chemotherapy as compared to other respective risk groups (*p* < 0.0001). Majority of the patients underwent subtotal colectomy (data not shown) and were least likely to receive adjuvant chemotherapy (19.9%) as compared to those who underwent partial (22.5%) or total (24.6%) colectomy.

### Comparison of Type of Adjuvant Chemotherapy by Age

Table 2 demonstrates differences of chemotherapy administration among various age groups stratified according to the type of chemotherapy. Young patients more often received chemotherapy, either single- or multi-agent, as compared to the elderly and the very elderly (11.3/25.3% vs. 8.2/11.9% vs. 3/2.4%). It is interesting to note that among the very elderly population, more patients received single-agent rather than multi-agent chemotherapy (3 vs. 2.4%).

**Table 2:** Modality of chemotherapy administered by age (chi-square test, *p* < 0.0001).

**Modality of chemotherapy**	**18–64 years, *n* (%)**	**65–74 years, *n* (%)**	**≥75 years, *n* (%)**	**Total (by chemotherapy, *N*)**
None	7,786 (59)	8033 (77.1)	18,167 (93.8)	33,986
Single agent	1,491 (11.3)	852 (8.2)	576 (3)	2,919
Multi agent	3,338 (25.3)	1,236 (11.9)	465 (2.4)	5,039
Type not documented	581 (4.4)	296 (2.8)	150 (0.8)	1,027
Total (by age group, *N*)	13,196	10,417	19,358	**42,971**

*Bold value means total sample size*.

### Determinant Factors for Adjuvant Chemotherapy Administration

In multivariate logistic regression analysis, patients with high-risk features were more likely to receive adjuvant chemotherapy (Table 3). Patients with pT4 lesions had higher likelihood of receiving chemotherapy compared to pT3 lesions (adjusted OR 3.54, 95% CI 3.27–3.34), as did patients with inadequate lymph node evaluation compared to those with adequate lymph node assessments (adjusted OR 1.15, 95% CI 1.08–1.22). High grade tumors were more likely to receive chemotherapy compared to well-differentiated tumors (adjusted OR 1.76, 95% CI 1.58–1.97) and patients with positive tumor margins had higher likelihood of receiving chemotherapy (adjusted OR 1.63, 95% CI 1.42–1.87). Young patients had higher odds of receiving adjuvant chemotherapy compared to very elderly (adjusted OR 11.69, 95% CI 10.84–12.6) and so did left sided tumors compared to right sided lesions (adjusted OR 1.27, 95% CI 1.19–1.35). Other significant factors associated with receipt of adjuvant chemotherapy included gender, race, comorbidity score, academic level of treating institution, geographic location and year of diagnosis.

**Table 3:** Adjusted odds ratios of adjuvant chemotherapy administration based on multivariate logistic regression.

	**Odds ratio**	**95% CI**	***p***
**Age**			
≥75 years	Ref = 1		
18–64 years	11.69	10.84–12.6	<0.0001
65–74 years	4.89	4.52–5.29	<0.0001
**Gender**			
Male	Ref = 1		
Female	1.06	1.0–1.12	0.0368
**Race**			
Black	Ref = 1		
White	1.11	1.02–1.21	0.0139
Hispanic	1.3	1.12–1.49	0.0003
Others	1.13	0.96–1.34	0.1337
**Charlson/deyo score**			
2	Ref = 1		
0	2.11	1.88–2.38	<0.0001
1	1.68	1.48–1.91	<0.0001
**Institution**			
Non-academic	Ref = 1		
Academic	1.19	1.12–1.27	<0.0001
**Location**			
Pacific	Ref = 1		
East North Central	1.4	1.26–1.55	<0.0001
East South Central	1.26	1.11–1.44	0.0005
Middle Atlantic	1.62	1.45–1.8	<0.0001
Mountain	1.05	0.9–1.23	0.5254
New England	1.16	1.01–1.34	0.0316
South Atlantic	1.15	1.04–1.27	0.006
West North Central	1.38	1.22–1.57	<0.0001
West South Central	1.2	1.06–1.36	0.0043
**Insurance**			
Uninsured	Ref = 1		
Insured	1.1	0.96–1.26	0.1694
**Income**			
< $30,000	Ref=1		
$30,000–$34,999	1.06	0.97–1.17	0.2071
$35,000–$45,999	0.98	0.9–1.08	0.7154
≥$46,000	1.01	0.93–1.11	0.7599
**Year of diagnosis**			
2009	Ref = 1		
2004	1.22	1.11–1.34	<0.0001
2005	1.24	1.13–1.36	<0.0001
2006	1.37	1.25–1.5	<0.0001
2007	1.29	1.18–1.41	<0.0001
2008	1.1	0.99–1.2	0.0526
**Primary site**			
Right	Ref = 1		
Left	1.27	1.19–1.35	<0.0001
Transverse	1.11	1.02–1.22	0.0194
**Grade**			
Well–differentiated	Ref = 1		
Moderately differentiated	1.14	1.04–1.25	0.0061
Poor/Undifferentiated	1.76	1.58–1.97	<0.0001
**Nodes evaluated**			
Adequate (≥ 12)	Ref = 1		
Inadequate (<12)	1.15	1.08–1.22	<0.0001
**AJCC, pT**			
3	Ref = 1		
4	3.54	3.27–3.34	<0.0001
**Colectomy**			
Total	Ref = 1		
Partial	1.08	0.93–1.26	0.3234
Subtotal	1.14	0.98–1.33	0.0797
**Margins**			
Negative	Ref = 1		
Positive	1.63	1.42–1.87	<0.0001

*Ref, Reference*.

### Independent Predictors of Overall Survival

**Figure 2:**
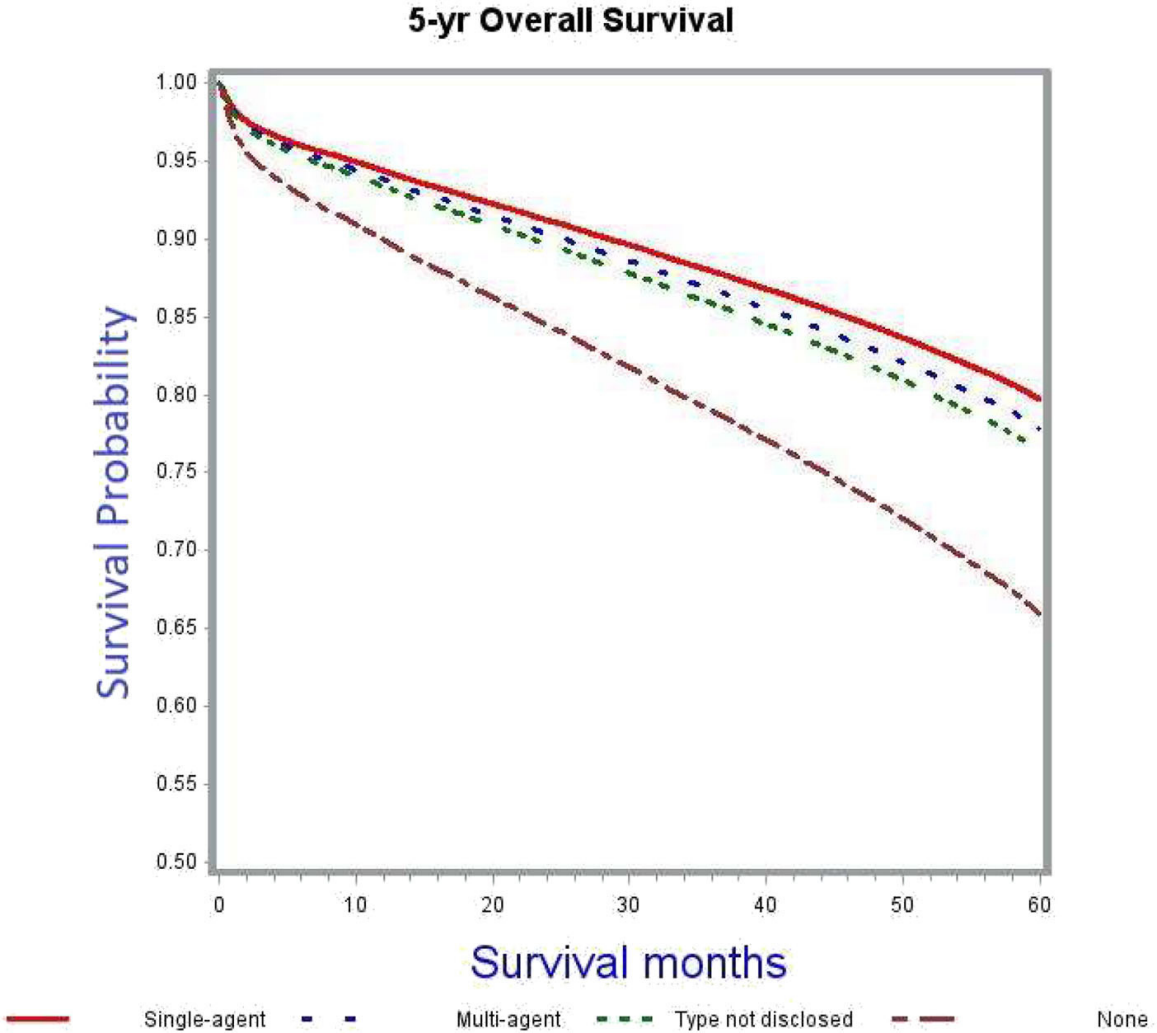
Adjusted survival curves stratified by adjuvant chemotherapy (*p* < 0.0001).

The crude 5-years OS rate for all patients receiving any kind of adjuvant chemotherapy was 73.5% as compared to 54.3% among those not receiving chemotherapy (Table 4). After adjusting for patient, tumor and treatment characteristics, the probability of death was significantly lower in patients receiving chemotherapy, irrespective of type and modality, as compared to patients not receiving adjuvant chemotherapy (single-agent: adjusted HR 0.55, 95% CI 0.51–0.59; multi-agent: adjusted HR 0.6, 95% CI 0.56–0.64; unknown type of chemotherapy: adjusted HR 0.65, 95% CI 0.57–0.73). Figure 2 demonstrates the association between adjuvant chemotherapy and improved OS. This further reflects the similar survivals associated with single- and multi-agent chemotherapy regimens in adjuvant setting.

**Table 4:** 5-years overall survival analysis using Cox proportional hazards regression model.

	**5-years survival, %**	**Hazards ratio**	**95% CI**	***p***
**Age**				
≥75 years	45.3	Ref = 1		
18–64 years	71.9	0.45	0.43–0.47	<0.0001
65–74 years	65.4	0.56	0.54–0.58	<0.0001
**Gender**				
Male	57.6	Ref = 1		
Female	59	0.86	0.83–0.88	<0.0001
**Race**				
Black	58.4	Ref = 1		
White	58.2	0.9	0.86–0.95	0.0001
Hispanic	58.1	1.03	0.94–1.12	0.5622
Others	63.4	0.95	0.86–1.06	0.3364
**Charlson/deyo score**				
2	39.2	Ref = 1		
0	62.6	0.58	0.55–0.61	<0.0001
1	53.7	0.7	0.68–0.74	<0.0001
**Institution**				
Non–academic	58.1	Ref = 1		
Academic	59	0.99	0.95–1.03	0.5834
**Location**				
Pacific	63.8	Ref = 1		
East North Central	58.9	1.16	1.1–1.24	<0.0001
East South Central	57.9	1.2	1.11–1.3	<0.0001
Middle Atlantic	54.5	1.32	1.24–1.4	<0.0001
Mountain	56.7	1.27	1.16–1.4	<0.0001
New England	56.4	1.15	1.07–1.24	0.0002
South Atlantic	58.7	1.16	1.1–1.23	<0.0001
West North Central	60.1	1.12	1.04–1.2	0.0037
West South Central	55.6	1.31	1.22–1.41	<0.0001
**Insurance**				
Uninsured	58.8	Ref = 1		
Insured	58.3	0.71	0.64–0.78	<0.0001
**Income**				
< $30,000	54.7	Ref=1		
$30,000–$34,999	56.6	0.95	0.9–0.99	0.0470
$35,000–$45,999	59.4	0.87	0.82–0.91	<0.0001
≥$46,000	61	0.85	0.81–0.89	<0.0001
**Year of diagnosis**				
2009	52.4	Ref = 1		
2004	59	0.79	0.75–0.83	<0.0001
2005	60.7	0.75	0.71–0.79	<0.0001
2006	59.7	0.78	0.74–0.82	<0.0001
2007	60.2	0.79	0.75–0.83	<0.0001
2008	57.9	0.86	0.81–0.9	<0.0001
**Primary site**				
Left	59	Ref = 1		
Right	58	0.93	0.9–0.97	0.0003
Transverse	58	0.95	0.9–1.00	0.0574
**Grade**				
Poor/Undifferentiated	55.2	Ref = 1		
Well-differentiated	60.1	0.88	0.82–0.93	<0.0001
Moderately diff	58.8	0.91	0.87–0.95	<0.0001
**Nodes evaluated**				
<12, inadequate	51.4	Ref = 1		
≥12, adequate	60.6	0.74	0.71–0.76	<0.0001
**AJCC pT**				
4	46.4	Ref=1		
3	59.6	0.59	0.57–0.62	<0.0001
**Chemotherapy**				
None	54.3	Ref = 1		
Single–agent	73.9	0.55	0.51–0.59	<0.0001
Multi–agent	73.6	0.6	0.56–0.64	<0.0001
Type not known	72.2	0.65	0.57–0.73	<0.0001
**Colectomy**				
Total	54	Ref = 1		
Partial	58.8	0.84	0.76–0.91	<0.0001
Subtotal	58.3	0.86	0.78–0.94	0.0005
**Margins**				
Positive	41.3	Ref = 1		
Negative	58.9	0.64	0.59–0.68	<0.0001

*Ref, Reference*.

**Figure 3:**
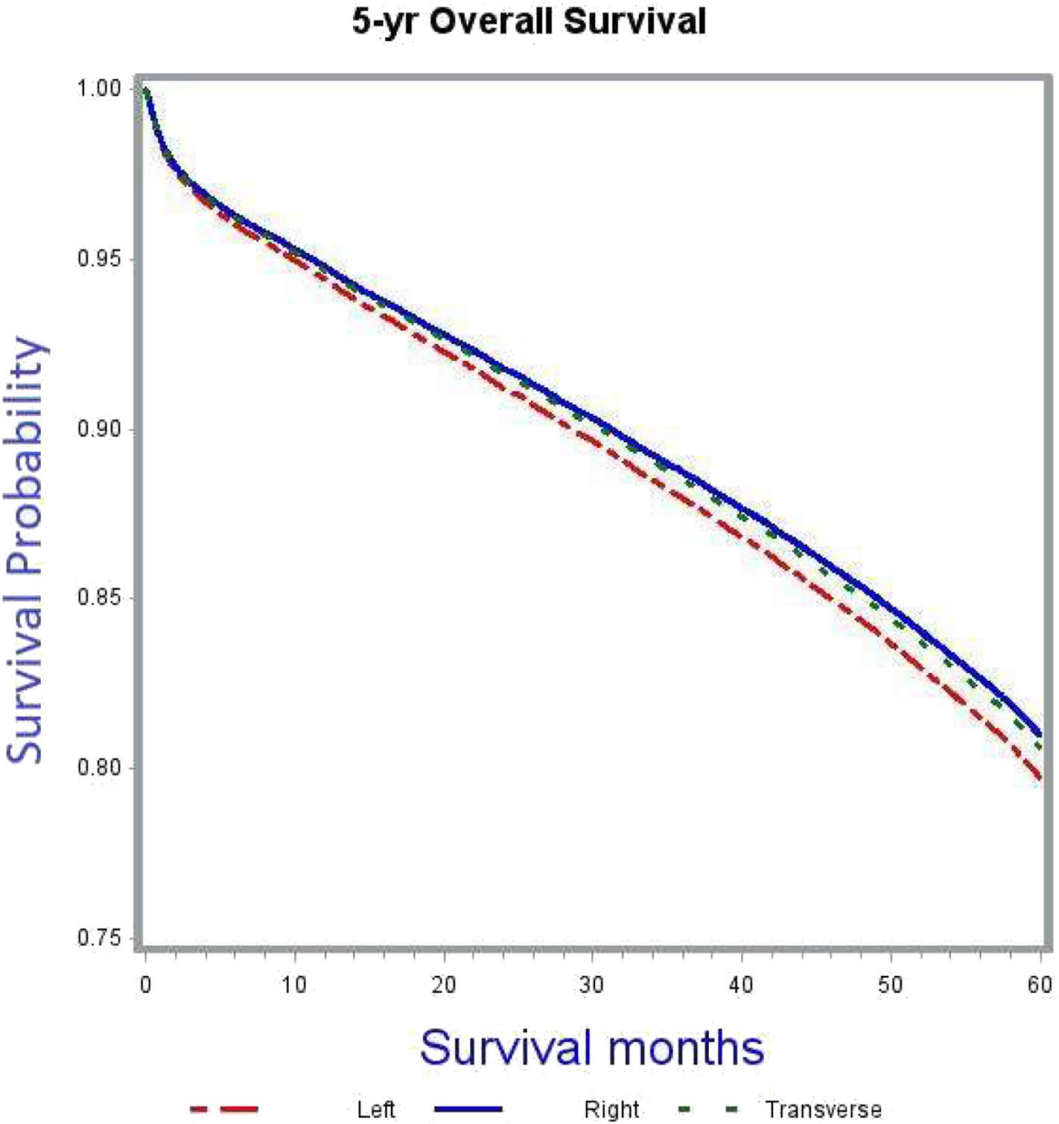
Adjusted survival curves stratified by side of primary tumor (*p* < 0.0001).

Patients with right sided tumors had better OS compared to left sided tumors (adjusted HR 0.93, 95% CI 0.9–0.97). Figure 3 demonstrates the association between tumor sidedness and improved OS. Among the four high-risk features evaluated in the study, including pT, grade, adequacy of lymph node evaluation and margins, improved OS was associated with pT3 lesions (adjusted HR 0.59, 95% CI 0.57–0.62, compared to pT4), low grade tumors (adjusted HR 0.88, 95% CI 0.82–0.93, compared to high grade tumors), adequate lymph node evaluation (adjusted HR 0.74, 95% CI 0.71–0.76, compared to inadequate lymph node assessment) and negative margins at surgery (adjusted HR 0.64, 95% CI 0.59–0.68, as compared to positive margins).

After adjusting for all other covariates, age, gender, ethnicity, Charlson/Deyo comorbidity index, geographic location, insurance status, median family income, year of diagnosis, and colectomy remained independent predictors of OS in stage II colon cancer (Table 4).

### Overall Survival Advantage of Adjuvant Chemotherapy by Tumor Sidedness

After adjusting for covariates, tumor sidedness was noted to demonstrate a significant survival benefit in thenon-chemotherapy subgroup (Table 5). In this subgroup right sided tumors had improved survival compared to left sided tumors (adjusted HR 0.92, 95% CI 0.88–0.96). This survival difference based on tumor location was however compensated with administration of chemotherapy, as demonstrated by the absence of significant OS benefit in the adjuvant chemotherapy subgroup analysis.

**Table 5:** Multivariate subgroup analysis of 5-years OS, according to adjuvant chemotherapy and tumor sidedness.

	**5-years survival, %**	**HR**	**95% CI**	***P***
**Adjuvant chemotherapy (*****n*** **=** **8,985)**				
Left sided	73.4	Ref = 1		
Right sided	73.5	1.04	0.94–1.15	0.4623
Transverse	74.1	0.96	0.83–1.13	0.6022
**No adjuvant chemotherapy (*****n*** **=** **33,986)**				
Left sided	54	Ref=1		
Right sided	54.6	0.92	0.88–0.96	<0.0001
Transverse	53.8	0.95	0.9–1.00	0.0677
**Left sided**				
**(*****n*** **=** **15,107)**				
No chemotherapy	54	Ref = 1		
Chemotherapy	73.4	0.58	0.53–0.62	<0.0001
**Right sided**				
**(*****n*** **=** **23,151)**				
No chemotherapy	54.6	Ref = 1		
Chemotherapy	73.5	0.6	0.56–0.65	<0.0001
**Transverse**				
**(*****n*****=** **4,713)**				
No chemotherapy	53.8	Ref = 1		
Chemotherapy	74.1	0.55	0.48–0.64	<0.0001

Furthermore, the OS benefit of adjuvant chemotherapy was confirmed in the subgroup analyses based on tumor sidedness. Irrespective of tumor location, adjuvant chemotherapy was associated with improved 5-years OS outcomes.

## Discussion

In stage II colon cancer, surgical resection is the mainstay of treatment with a wide 5-years OS range which highlights the heterogeneity that exists among stage II colon cancers in term of recurrence. The survival benefit of adjuvant chemotherapy in stage III patients is well-established but a definitive benefit in stage II patients remains unclear (8–16, 22–24, 28–30). Some of these studies have demonstrated disease specific and/or overall survival benefit of adjuvant chemotherapy in stage II colon cancers with high-risk features. Current clinical practice guidelines and the consensus statement from the recent Cochrane review recommend discussion of adjuvant chemotherapy with stage II colon cancer patients having high risk features (19, 20, 31). As a result, there is a wide variation regarding the decision to administer adjuvant chemotherapy among individual physicians, institutions, and countries.

The current study was undertaken to provide a better assessment of the OS benefit of adjuvant chemotherapy in stage II colon cancer with regard to various high-risk features including pT4, inadequate lymph node evaluation, high grade tumors and those with positive surgical margins, along with the other important prognostic factor of tumor sidedness (which was considered as a surrogate for MSI status) (32, 33). We found that after controlling for various patient, tumor, and treatment characteristics, adjuvant chemotherapy had a significant OS benefit in stage II colon cancer. This is consistent with the findings of earlier studies including the QUASAR trial (12) and a retrospective analysis by Casadaban et al. (24). Single-agent chemotherapy fared as well as multi-agent chemotherapy when compared to no adjuvant chemotherapy (Figure 2). Though the overall use of adjuvant chemotherapy decreased with advancing age; the elderly and very elderly patients were more likely to receive single-agent chemotherapy (Table 2) compared to young patients. Despite this difference, the outcomes favored adjuvant chemotherapy, which is in line with findings from the ACCENT database and the MOSAIC trial (9, 34).

In the multivariate Cox proportional hazards model analysis, patients with right sided tumors had better OS compared to left sided tumors. A previous retrospective analysis by Weiss et al. (30) using the Surveillance, Epidemiology and End Results (SEER)-Medicare data showed no OS benefit of adjuvant chemotherapy for either right- or left-sided tumors. However, this study included only Medicare beneficiaries aged 66 years and older. To further delineate into the survival interactions between tumor sidedness and adjuvant chemotherapy in our study cohort, a set of subgroup analyses was carried out. In the subgroup not receiving any adjuvant chemotherapy right sided tumors had better OS outcomes compared to left sided tumors (adjusted HR 0.92, 95% CI 0.88–0.96). This difference was compensated for in the subgroup receiving adjuvant chemotherapy (Table 5). Right-sided tumors clinically correlate for MSI-H status and based on available evidence of MSI-H tumors not responding to 5-FU based adjuvant chemotherapy (21), it could be argued that administration of chemotherapy in these tumors could have resulted in worsening of survival outcomes thereby nullifying the survival difference between right- and left-sided tumors. This was further put to test through additional subgroup analyses of left-sided, right-sided, and transverse colon only cancers. In these multivariate Cox proportional regression analyses, administration of chemotherapy resulted in significant OS benefit for all tumor locations (adjusted HR and 95% CI: left-sided 0.58, 0.53–0.62; right-sided 0.6, 0.56–0.65; transverse 0.55. 0.48–0.64). These results support the benefit of adjuvant chemotherapy irrespective of tumor location.

High-risk features in stage II colon cancer traditionally included pT4, tumor perforation or bowel obstruction, high grade or poorly differentiated tumors, lympho-vascular invasion and <12 lymph nodes examined. According to the current guidelines, it is recommended to discuss adjuvant chemotherapy with patients having any one or combination of these risk factors (19, 20). Using the current study cohort, we evaluated the odds of adjuvant chemotherapy administration, from 2004 to 2009, based on some of these high-risk features that were available through NDCB. These included pT4, high-grade tumors and examination of <12 lymph nodes. As depicted in Table 3, patients with pT4 lesions were more likely to receive chemotherapy than those with pT3 lesions. Verhoeff et al. (23) analyzed data from the Netherlands Cancer Registry and found improved survival outcomes with adjuvant chemotherapy in patients with pT4 stage II colon cancer. Tumors with undifferentiated histology or higher-grade were more likely to receive adjuvant chemotherapy. This is in contrary to the idea of poorly differentiated tumors being clinically correlated to MSI-H status, which portends a poor response to 5-FU based chemotherapy (21, 35). Inadequate lymph node evaluation was shown to carry a poor prognostic effect on OS in a recent study by Reha et al. (36) using the NCDB. On the same note, patients with inadequate lymph node evaluation in our study cohort were more likely to receive adjuvant chemotherapy to help improve the odds of OS attributable to disease recurrence.

There are several limitations to this study. First, this is a non-randomized, retrospective analysis that allows for a potential selection bias. Second, information on the type of chemotherapy regimen, adherence and completion rates were not available in the NCDB. This creates a heterogeneous population in which patients could receive substandard duration of therapy. Lack of information on the type of chemotherapy regimen was partially compensated by the availability of data on single vs. multi-agent chemotherapy. Analysis of certain data variables was restricted by availability in the NCDB file, including MSI status, disease specific mortality, colonic obstruction or perforation and missing information from the lympho-vascular invasion data collection.

Despite these limitations, the current study is the largest population-based analysis of stage II colon cancer only patients in the U.S. Though the previous study by Casadaban et al. (24) included nearly 4-times the number of patients in our study, their cohort included stage II colon cancer patients with other malignancies, who were excluded from our study cohort. Our study population included adult patients belonging to all age groups as compared to the SEER-Medicare study by Weiss et al. (30) that included patients aged 66 years and older.

## Conclusion

The results of our study suggest the use of adjuvant chemotherapy in stage II colon cancer. There was a statistically significant 5-years OS benefit seen after adjusting for available patient, tumor and treatment characteristics including high-risk features as well as tumor location. Subgroup analysis further confirmed the survival benefit associated with adjuvant chemotherapy irrespective of tumor sidedness. However, owing to the observational nature of this study, interpretation and clinical application should be undertaken with caution. Future validation with prospective trials including MSI status is warranted.

## Data Availability Statement

The raw data supporting the conclusions of this article will be made available by the authors, without undue reservation.

## Disclosure

This study was presented in poster format at the ASCO Gastrointestinal Cancers Symposium; January, 2017; San Francisco, California.

## Author Contributions

RR and SM had full access to all the data in the study and take responsibility for the integrity of the data and the accuracy of the data analysis, acquisition, analysis, or interpretation of data, and administrative, technical, or material support. SM and DH drafting of the manuscript. PS and RR critical revision of the manuscript for important intellectual content. SM statistical analysis. RR study supervision. All authors study concept and design.

## Conflict of Interest

The authors declare that the research was conducted in the absence of any commercial or financial relationships that could be construed as a potential conflict of interest.

## Abbreviations

OS: Overall survival
ASCO: American Society of Clinical Oncology
NCCN: National Comprehensive Cancer Network
MSS: microsatellite stability
NCDB: National Cancer Data Base
FDA: Food and Drug Administration
FOLFOX, 5-fluorouracil: leucovorin and oxaliplatin
PUF: Participant User File
AJCC: American Joint Committee on Cancer
CCS: Collaborative Stage Data Collection System
OR: odds ratio
HR: hazards ratio
CI: confidence interval
MSI: micro-satellite instability
SEER, Surveillance: Epidemiology and End Results.
