# Supplementary figures and images for: Adjuvant Chemotherapy and Tumor Sidedness in Stage II Colon Cancer: Analysis of the National Cancer Data Base
Source: Front Oncol. 2020 Sep 15;10:568417. doi: 10.3389/fonc.2020.568417 (PMC7523086; doi:10.3389/fonc.2020.568417)

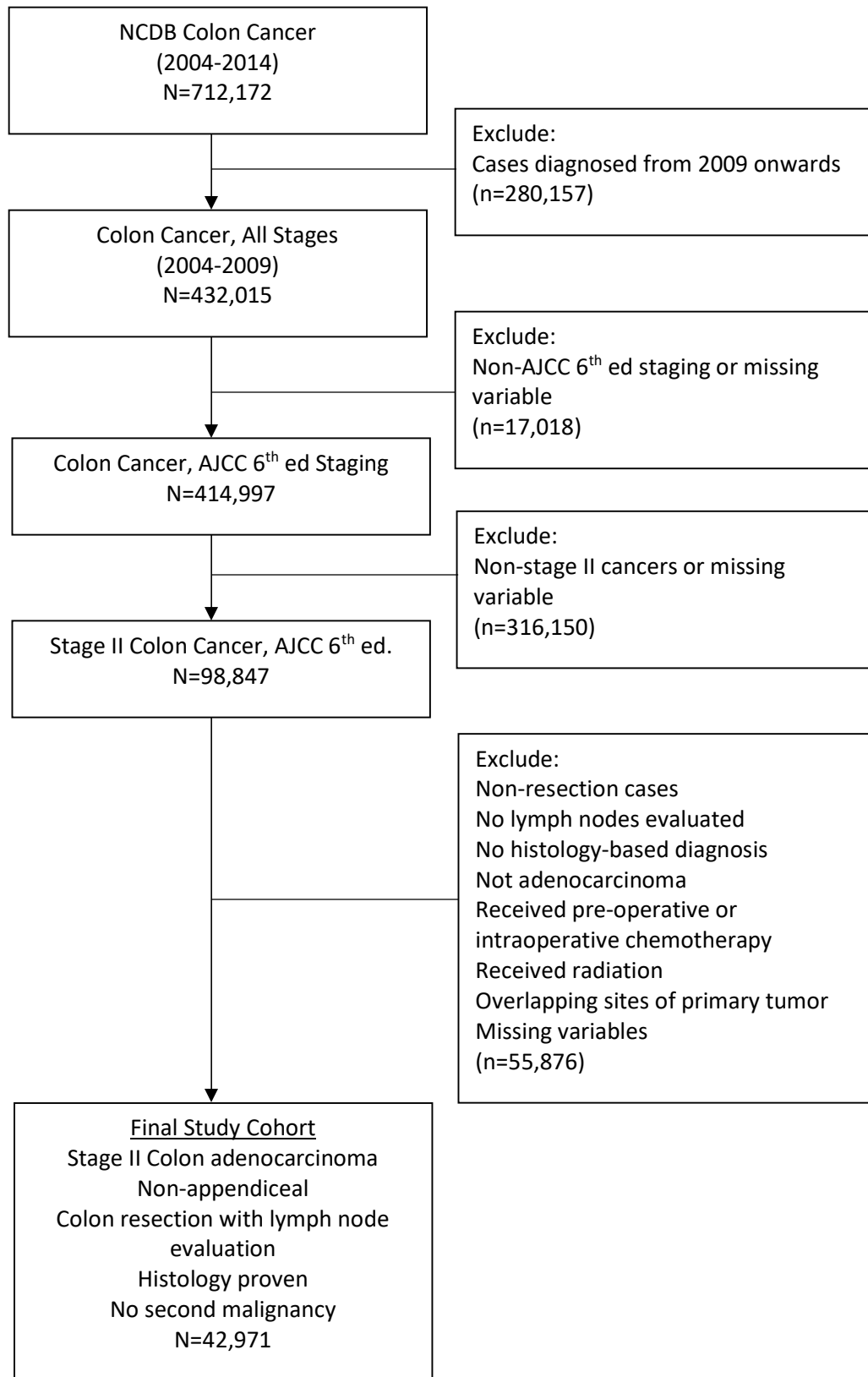

Supplement: Supplementary file 1 [file Data_Sheet_1.PDF]
